# Supplementary material for: Modulatory Effect of Cucurbitacin D from Elaeocarpus hainanensis on ZNF217 Oncogene Expression in NPM-Mutated Acute Myeloid Leukemia
Source: Pharmaceuticals (Basel). 2024 Nov 21;17(12):1561. doi: 10.3390/ph17121561 (PMC11676938; doi:10.3390/ph17121561)
Supplement: Supplementary file 1 [file pharmaceuticals-17-01561-s001.zip › pharmaceuticals-3207047-supplementary.pdf]

Table of Contents

	Page
**Materials and methods**<br>**General experimental procedures**	2
**Table S1**. ^{13}C (125 MHz) and ^{1}H (500 MHz) NMR data of compounds **1- 3**	3
**Figure S1.** ^{1}H NMR (500 MHz, $CDCl_3$) spectrum of CucD (**1**)	4
**Figure S2.** ^{13}C NMR (125 MHz, $CDCl_3$) spectrum of CucD (**1**)	5
**Figure S3.** ^{1}H NMR (500 MHz, CD_3OD) spectrum of IsocucD (**2**)	5
**Figure S4.** ^{13}C NMR (125 MHz, CD_3OD) spectrum of IsocucD (**2**)	6
**Figure S5**. DEPT-^{13}C NMR (125 MHz, CD_3OD) spectrum of IsocucD (**2**)	6
**Figure S6.**^{1}H NMR (500 MHz, CD_3OD) spectrum of mixture CucD + CucI (**3**)	7
**Figure S7.**^{13}C NMR (125 MHz, CD_3OD) spectrum of mixture CucD + CucI (**3**)	8
**Figure S8.** DEPT-^{13}C NMR (125 MHz, CD_3OD) spectrum of mixture CucD + CucI (**3**)	8

Materials and methods

General experimental procedures

Columnchromatography (CC) was performed using silica gel (40-63 µm) or reversed phase C-18 (RP18, 150 µm) and Sephadex LH-20asadsorbents. Thin-layer chromatography (TLC) was carried out on pre-coated silica gel 60 F_{254}. For spot visualization, UV light and a vanillin-H_2SO_4 reagent were used.

NMR spectra were recorded using a Bruker Avance-500 spectrometer operating at frequencies of 500 MHz (^{1}H) and 125 MHz (^{13}C). The spectra were measured in CD_3OD. ^{1}H and ^{13}C NMR chemical shifts (δ) were expressed in ppm with reference to solvent signal [$CDCl_3$: δH 7.27 and δC 77.3; CD_3OD: δH 3.33 and δC 49.0].

Plant material

The plant materials were collected in Ha Tinh province, Vietnam, in November 2021. *Elaeocarpus hainanensis*Oliv. species was botanically certified by Dr. Do Ngoc Dai, Department of Forestry, Nghe An University of Economics, and a voucher specimen (No. 04TN.EH21) was deposited in the Institute of Chemistry, VAST.

Extraction and isolation of cucurbitacins.

Dried twigs and leaves of *E.hainanensis* Oliv. (5.0 kg) were cut into pieces and extracted with methanol (MeOH) at room temperature, filtered, and the solvent was removed under

reduced pressure to give a crude MeOH extract (285g). This extract was suspended with water and sequentially partitioned with *n*-hexane and dichloromethane (CH_2Cl_2) to yield corresponding soluble fractions *n*-hexane (76 g), dichloromethane (ED, 60 g), and a residual water layer. Fraction ED was roughly chromatographed on a silica gel column, using a gradient solvent system of CH_2Cl_2-MeOH, 9/1→5/5, v/v) to obtain ten fractions ED1 – ED10. Fraction ED4 (10 g) was continuously separated on a silica gel column by using a gradient of CH_2Cl_2–acetone (1/9→5/5, v/v) to give 4 subfractions (ED4.1- ED4.4). Subfraction ED4.2 (1.15 g) was purified by a SiO_2 gel chromatography column using a gradient of CH_2Cl_2–acetone (5/5, v/v) to afford compound **1** (21 mg). Fraction ED5 (12 g) was re-chromatographed on a SiO_2 gel column using CH_2Cl_2–acetone (9/9→1/9, v/v) as an eluent to obtain six subfractions (ED5.1 – ED5.6). Subfraction ED5.2 (320 mg) was further purified by repeated column chromatography over Sephadex LH-20 to yield compound **2** (12 mg) and mixture compounds **1** and **3** (10 mg), respectively. The spectroscopic data for each isolate were compared to previously reported literature, including NMR(Table S1), HR ESI MS data: Cucurbitacin D (CucD, **1**), 3-*epi*-isocucurbitacin D (isocucD, **2**), and mixture of cucurbitacin D and cucurbitacin I (CucD + CucI, **1** + **3**).The ratio of compounds **1** and **3** in the mixture (**1**:**3** =1:1) is determined by the protonintegralintensity (H-6).

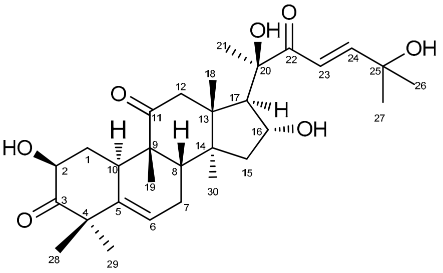

1: Cucurbitacin D (CucD)

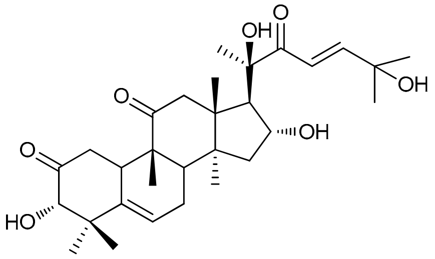

2: 3-*epi*-Isocurbitacin D (Isocuc D)

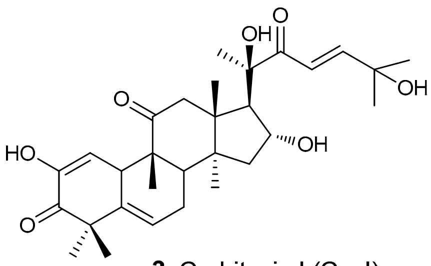

3: Curbitacin I (CucI)

Table S1. ^{13}C (125 MHz) and ^{1}H (500 MHz) NMR data of compounds **1**- **3**

C	**1,** $CDCl_3$		**2,** CD_3OD		**3,** CD_3OD	
	δ_C	δ_H	δ_C	δ_H	δ_C	δ_H
1	36.0	2.32 ddd (9.0, 6.0, 3.5)<br>1.21, d (7.0)	38.0	2.25 m, 2.21. m	116.7	5.99. d (2.5)
2	71.6	4.45 dd (6.0, 13.0)	213.0	-	146.8	-
3	213.0	-	81.2	4.10 s	216.1	-
4	50.3	-	42.5	-	47.4	-
5	140.5	-	141.1	-	140.0	-
6	120.3	5.78 dd (3.5,2.5)	122.9	5.92 t (5.2)	121.3	5.82 m
7	23.9	1.95 m<br>2.40 ddd (17.0, 5.5. 2.5)	24.8	2.05 m	24.6	1.94 m<br>2.40 m
8	42.4	1.97 m	44.2	2.01 m	43.3	2.07 m
9	48.3	-	49.5	-	48.8	-
10	33.8	2.78 br d (13.0)	34.1	3.13 m	35.9	2.86 m
11	212.2	-	215.7	-	212.0	-
12	48.7	3.31 d (14.5)<br>2.69 d (14.5)	49.9	2.59 m,<br>3.33 d (13.8)	48.6	3.42 d (14.5)<br>2.67d (14.5)
13	50.8	-	51.7	-	50.2	-
14	48.4	-	49.3	-	48.3	-
15	45.5	1.37 m,<br>1.87 dd (13.0, 8.5)	46.8	1.46m,<br>1.87 dd (12.0, 9.0)	46.6	1.41 m<br>1.89 m
16	71.5	4.34 t (7.3)	71.5	4.50 t (7.5)	71.7	4.50 m
17	57.3	2.56 d (7.3)	59.5	2.57 m	59.5	2.50 d (6.0)
18	20.0	0.98 s	20.7	0.92 s	20.3	0.95 s
19	20.1	1.08 s	19.4	1.09 s	20.7	1.01 s
20	78.1	-	79.9	-	79.9	-
21	24.0	1.39s	25.4	1.40 s	24.7	1.40 s
22	202.5	-	205.0	-	204.9	-
23	119.0	6.63 d (15.3)	121.3	6.84 d (15.5)	121.7	6.85d (15.2)
24	155.9	7.13 d (15.3)	155.3	6.98 d (15.5)	155.4	7.00 d (15.2)
25	71.2	-	71.7	-	71.7	-
26	29.5	1.35 s	29.2	1.34 s	29.3	1.39 s
27	29.3	1.35 s	29.2	1.35 s	29.1	1.33 s
28	28.9	1.30 s	19.0	1.41 s	28.3	1.34 s
29	21.3	1.34 s	24.7	0.96 s	21.6	1.29 s
30	19.3	1.34 s	27.6	1.24 s	18.9	1.34 s

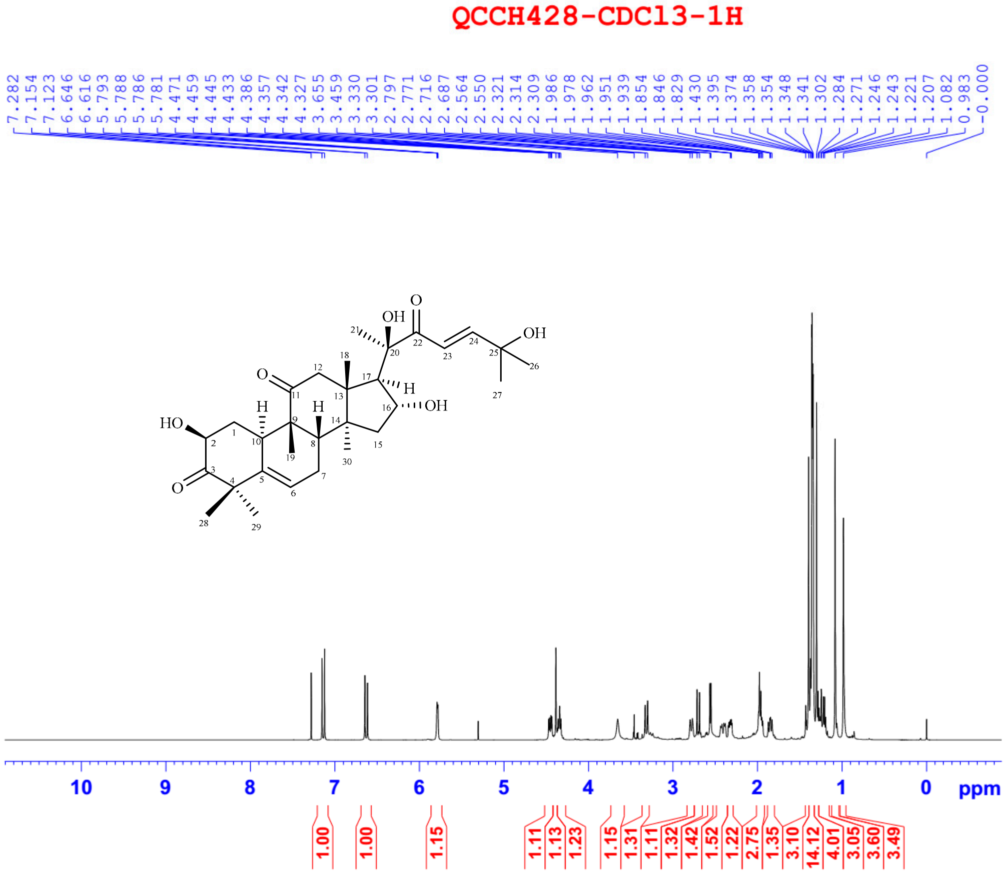

Figure S1. ^{1}H NMR (500 MHz, $CDCl_3$) spectrum of CucD (**1**)

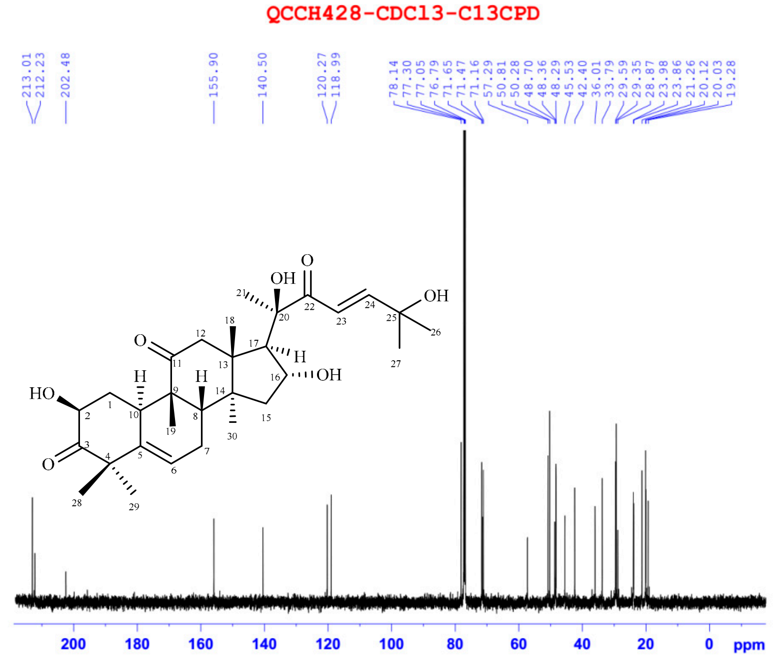

Figure S2. ^{13}C NMR (125 MHz, $CDCl_3$) spectrum of CucD (**1**)

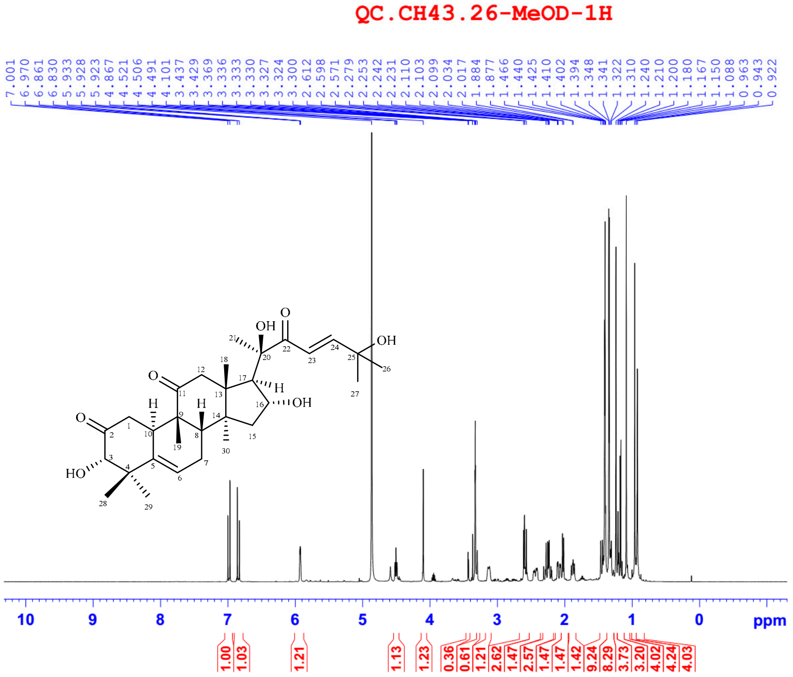

Figure S3. ^{1}H NMR (500 MHz, CD_3OD) spectrum of IsocucD (**2**)

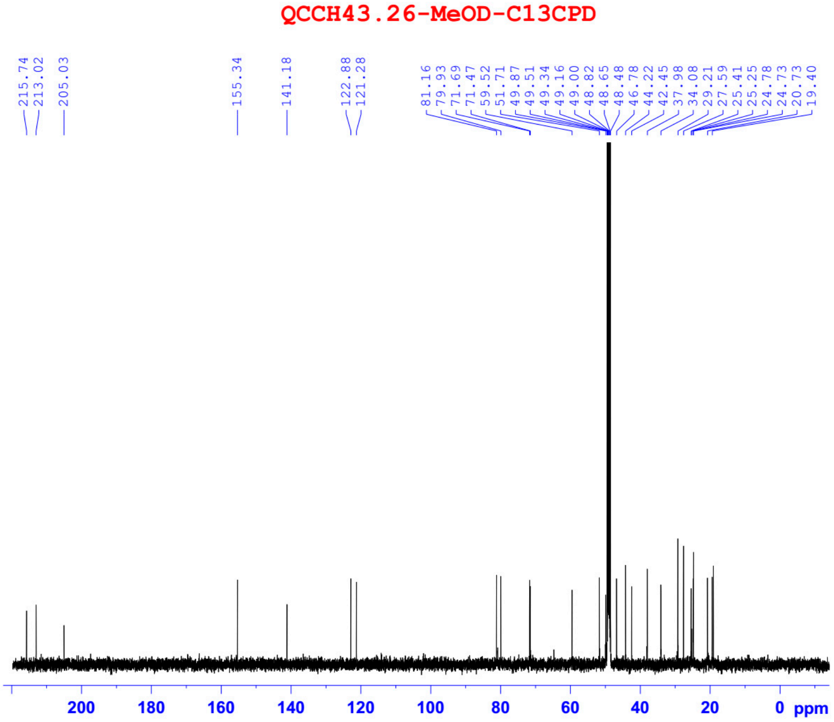

Figure S4. ^{13}C NMR (125 MHz, CD_3OD) spectrum of *Iso*cucD (**2**)

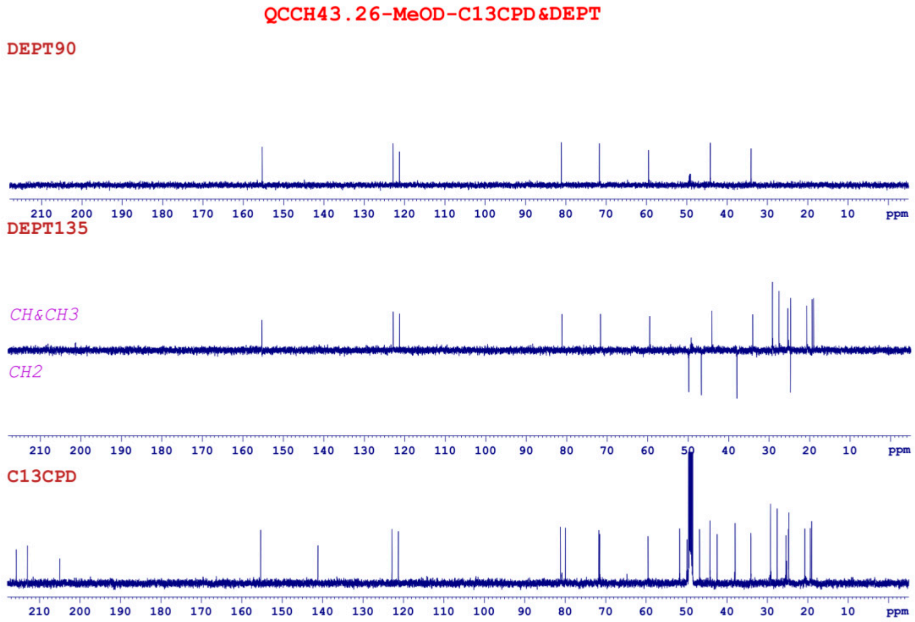

Figure S5. DEPT-^{13}C NMR (125 MHz, CD_3OD) spectrum of IsocucD (**2**)

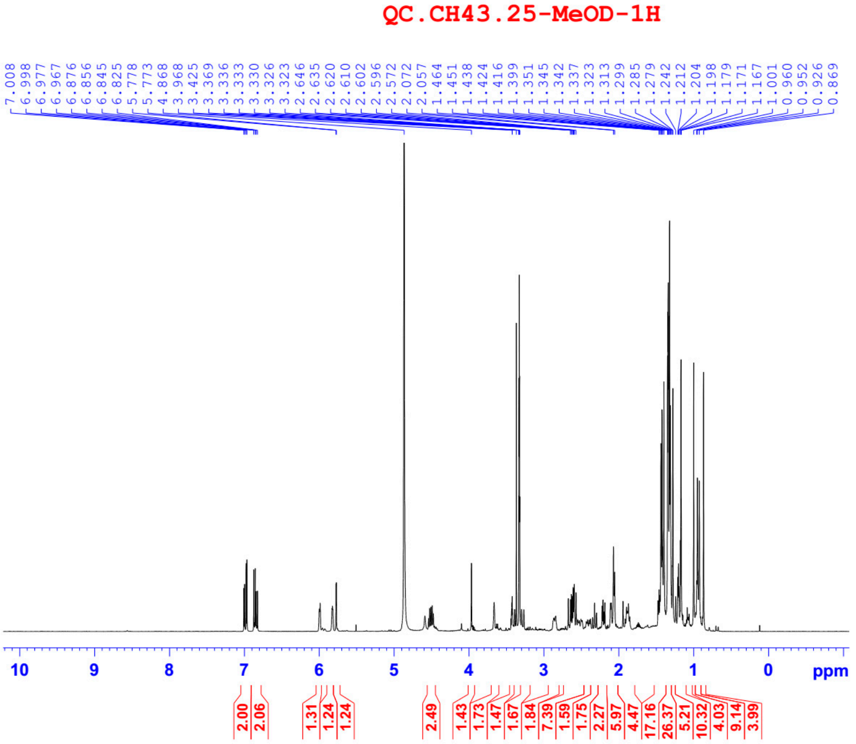

Figure S6.^{1}H NMR (500 MHz, CD_3OD) spectrum of mixture CucD + CucI (**3**)

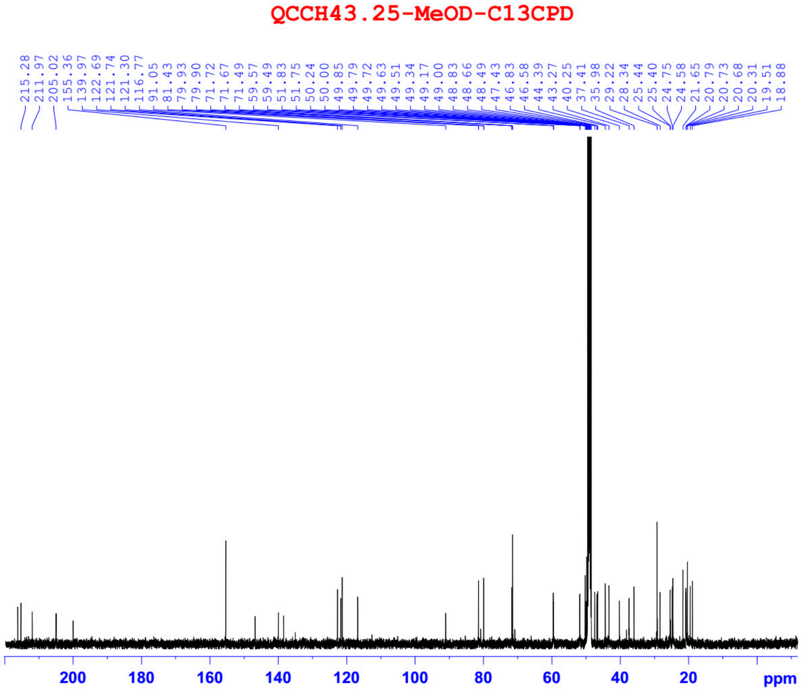

Figure S7. ^{13}C NMR (125 MHz, CD_3OD) spectrum of mixture CucD + CucI (**3**)

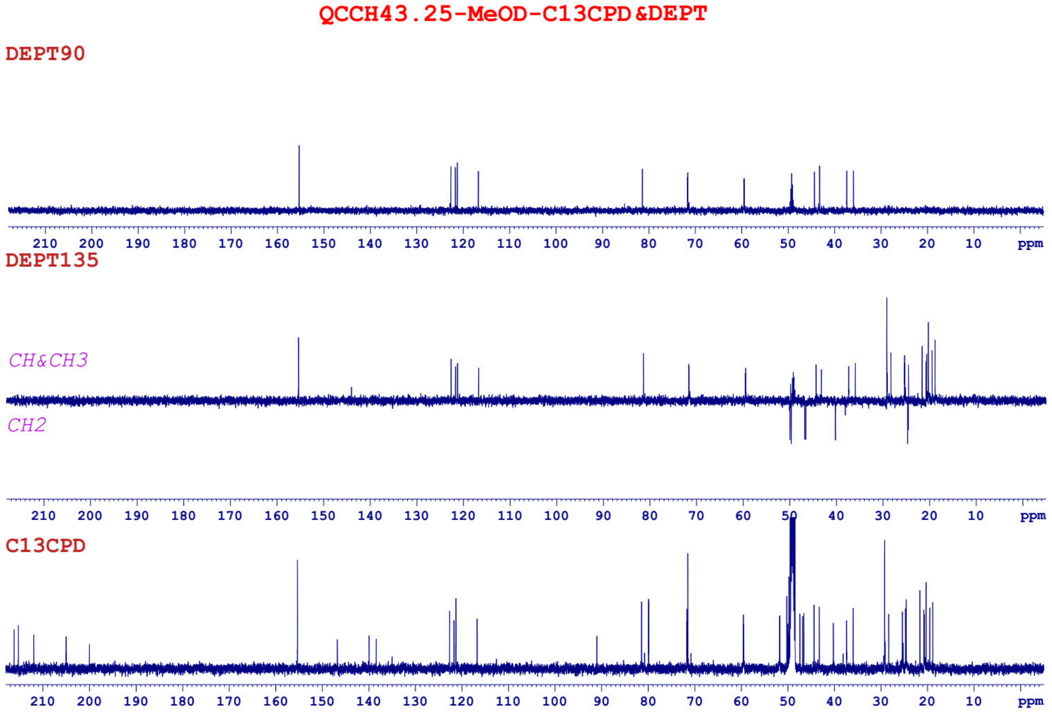

Figure S8. DEPT-^{13}C NMR (125 MHz, CD_3OD) spectrum of mixture CucD + CucI (**3**)
